# Elastofibroma dorsi – differential diagnosis in chest wall tumours

**DOI:** 10.1186/1477-7819-5-15

**Published:** 2007-02-05

**Authors:** Adrien Daigeler, Peter Maria Vogt, Kay Busch, Werner Pennekamp, Dirk Weyhe, Marcus Lehnhardt, Lars Steinstraesser, Hans-Ulrich Steinau, Cornelius Kuhnen

**Affiliations:** 1 Department of Plastic Surgery, Burn Center, Hand Center, Sarcoma Reference Center, BG-Hospital "Bergmannsheil", Ruhr-University Bochum, Bürkle-de-la-Camp-Platz 1, 44789 Bochum, Germany; 2 Department of Plastic, Hand, and Reconstructive Surgery, Burn Center, Hannover Medical School, Podbielskistr. 380, 30659 Hannover, Germany; 3 Institute of Diagnostic Radiology, Interventional Radiology and Nuclear Medicine, BG-Hospital "Bergmannsheil", Ruhr-University Bochum, Bürkle-de-la-Camp-Platz 1, 44789 Bochum, Germany; 4 Department of Surgery, St. Josef Hospital – University Medical Center, Ruhr University of Bochum, Gudrunstr. 56, 44791Bochum, Germany; 5 Pathology, BG-Hospital "Bergmannsheil", Ruhr-University Bochum, Bürkle-de-la-Camp-Platz 1, 44789 Bochum, Carl-Neuberg-Str. 1, 39625 Hannover, Germany

## Abstract

**Background:**

Elastofibromas are benign soft tissue tumours mostly of the infrascapular region between the thoracic wall, the serratus anterior and the latissimus dorsi muscle with a prevalence of up to 24% in the elderly. The pathogenesis of the lesion is still unclear, but repetitive microtrauma by friction between the scapula and the thoracic wall may cause the reactive hyperproliferation of fibroelastic tissue.

**Methods:**

We present a series of seven cases with elastofibroma dorsi with reference to clinical findings, further clinical course and functional results after resection, as well as recurrence. Data were obtained retrospectively by clinical examination, phone calls to the patients' general practitioners and charts review. Follow-up time ranged from four months to nine years and averaged 53 months.

**Results:**

The patients presented with swelling of the infrascapular region or snapping scapula. In three cases, the lesion was painful. The ratio men/women was 2/5 with a mean age of 64 years. The tumor sizes ranged from 3 to 13 cm. The typical macroscopic aspect was characterized as poorly defined fibroelastic soft tissue lesion with a white and yellow cut surface caused by intermingled remnants of fatty tissue. Microscopically, the lesions consisted of broad collagenous strands and densely packed enlarged and fragmented elastic fibres with mostly round shapes. In all patients but one, postoperative seroma (which had to be punctuated) occurred after resection; however, at follow-up time, no patient reported any decrease of function or sensation at the shoulder or the arm of the operated side. None of the patients experienced a relapse.

**Conclusion:**

In differential diagnosis of soft tissue tumors located at this specific site, elastofibroma should be considered as likely diagnosis. Due to its benign behaviour, the tumor should be resected only in symptomatic patients.

## Background

Elastofibromas are slowly growing benign tumors of soft tissue origin. In 99% of the cases, they are located in the inferior subscapular region between the scapula and the thoracic wall; they consist of fragmented and enlarged elastic fibres embedded in collagenous matrix that often occur bilaterally [1-4]. They are commonly found in active subjects beyond their 50^th ^year [1-4], but may also affect children [5]. In elderly patients, this tumor was incidentally found in up to 2% by CT imaging [6]. Autopsy studies reported an even higher incidence of 13% to 17%, revealing pre-elastofibroma-like morphologic changes even in 81% of the autopsies [7,8]. In subjects over 55 years of age, prevalence is given with up to 24% [8]. In the differential diagnosis of soft tissue tumors located at the thoracic wall, one should be aware of this surprisingly common lesion. We followed up a series of seven cases with reference to clinical findings, further clinical course and functional result after resection, as well as recurrence.

## Patients and methods

Data for this case series were acquired retrospectively from the patients' charts, physical examination and phone calls to their general practicioners. From 1996 to 2005, seven patients with diagnosis elastofibroma dorsi were treated at our institutions (two center study).

All patients underwent MRI imaging preoperatively. Three patients were core biopsied in advance; for two patients, the diagnosis was made by instantaneous section. Two patients were primarily resected completely without pre- or intraoperative histopathologic verification of the diagnosis. In all patients, marginal complete resection was performed and tissue specimens were sent in for pathological evaluation to an experienced soft tissue pathologist. Defects were closed primarily.

## Results

Five patients were female; two were male. The average age at the time of the treatment was 63.7 years and ranged from 46 to 79 years. Follow-up data was available for all patients. Follow-up time ranged from four months to nine years and averaged 53 months.

**Figure 1:**
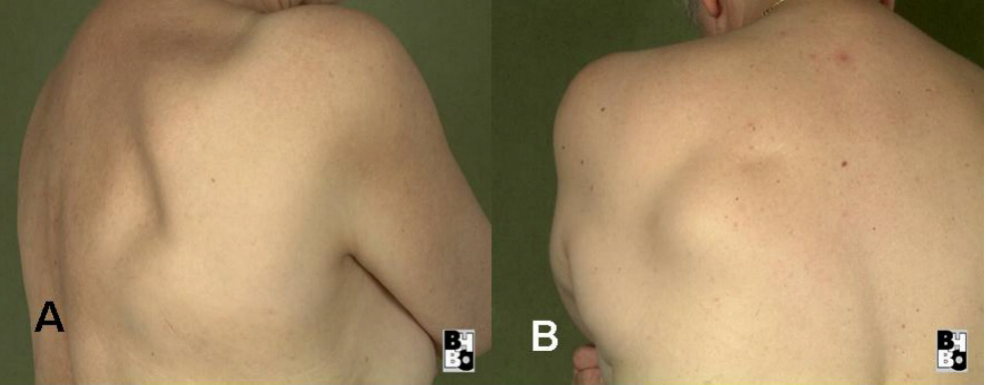
Photographs of the tumour aspect of patients 1 (a) and 1 (b).

**Figure 2:**
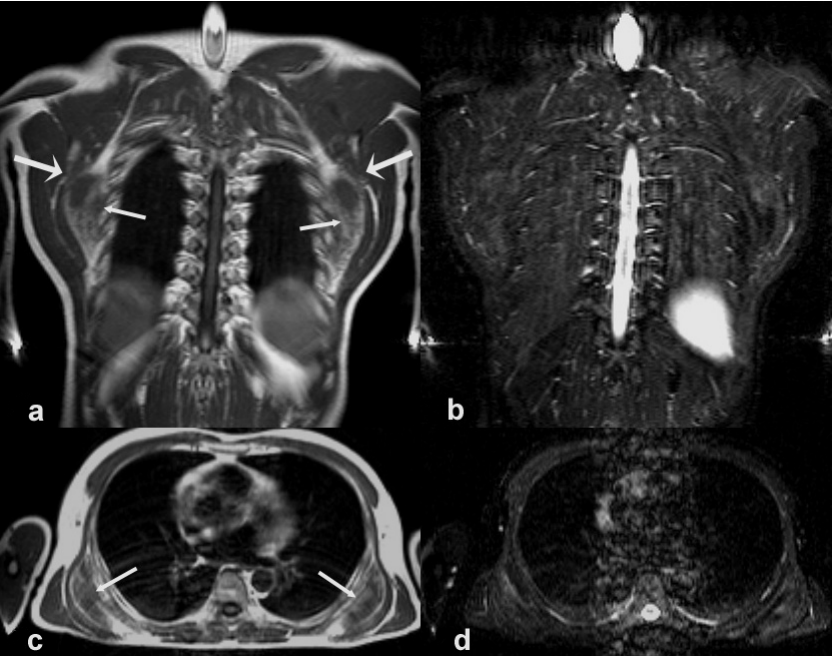
MRI of bilateral elastofibroma with the tumour being located between the thoracic wall, the anterior serratus, and the latissimus dorsi muscle (coronal (2a) and axial (2c) T1-weighted images): The small arrows indicate the medial margins of the lesions containing fatty (bright) and fibrous (dark) tissue. The tumours are located between the thoracic wall, the anterior serratus, and the latissimus dorsi muscle. The large arrows points to the margo inferior of the scapula. Figures [2b] and [2d] show the corresponding STIR -sequences with a slightly inhomogenous signal intensity within the elastofibromas.

The tumors were attached to the thoracic wall, located between the serratus anterior muscle and the chest wall in their anterior and between serratus and latissimus dorsi muscle in their posterior extension (figure 1). In four cases, the tumor was located at the right side; in two cases, at the left side; and, in one patient, it was growing bilaterally. In only three cases, the lesion was located at the side of the dominant hand. None of the patients was extensively active during their lifetime. In most of the patients, the predominant symptoms were swelling and snapping of the scapula. The tumor sizes ranged from 3 to 13 cm. On MRI in all patients the margins of the elastofibromas were well defined. The tumors were located inferior to the margo inferior of the scapula, adjacent to the thorcic wall. The central parts contained fibrous masses with low signal intensity on T1- and T2-weighted images. Hyperintens signals in T1- and T2- weighted images represented intermingled fatty tissue. On STIR-sequences the fatty tissue also showed low signal intensity, with a slightly higher intensity of the the fibrous tissue. Focal aereas of high signal intensity STIR-sequences may also be interpreted as edema within the lesion. In figure 2 the typical aspect of a bilateral elastofibroma on MRI is shown (figure 2).

In one patient, dyspnea and hypertension occurred coincidentally with the elastofibroma and disappeared with tumor resection. Due to the mild disturbances caused by the lesions, the patients sought medical advice only late after the onset of the symptoms (4 months to 4 years). In all patients but one, postoperative seroma (which had to be punctuated) occurred; however, at follow-up time, no patient reported any decrease of function or sensation at the shoulder or the arm of the operated side. No relapse of the tumor occurred. One patient could not be followed up personally as the patient died of cerebral apoplexy 13 months after treatment. At that time, however, the patient had neither recurrence of the elastofibroma nor any disturbances caused by the tumor resection. A detailed summary of the patients' data is given in table 1.

**Table 1:** Detailed summary of patient data

**Patient**	**Age**	**Gender**	**Profession**	**Handedness**	**Location**	**Tumor extension in cm**	**Symptom**	**Comorbidity**	**Duration of symptoms before treatment**	**Operative procedure**	**Recurrence**	**Complication**	**Disturbances at follow up**
1	68	F	housewife	right	right subscapular region between serratus and latissimus muscle	6x6x4	swelling	hypertension	4 years	complete marginal resection	no	seroma	none
2	76	F	housewife	right	right subscapular region between serratus and latissimus muscle	12x7x5	pain	coronary artery disease, diabetes mellitus IIb	9 months	complete marginal resection	no	seroma	none, died of cerebral apoplexy 13 months after treatment
3	46	F	nurse	right	left subscapular region between serratus and latissimus muscle	5.5x5x2	swelling	hypertension, hyperthyreosis	4 months	complete marginal resection	no	seroma	none
4	71	F	housewife	left	right subscapular region between serratus and latissimus muscle	3x3x3	swelling	Leiomyosarcoma both lower leg,s gonarthrosis	2 years	complete marginal resection	no	none	none
5	47	M	engineer	right	left subscapular region betweeen latissismus and serratus muscle	5x4x2	pain	hypertension	4 years	complete marginal resection	no	hematoma	none
					right subscapular region between latissimus, serratus, and rhomboideus maj. muscle	13x11x3	pain		4 years	complete marginal resection	no	seroma	none
6	59	M	engineer	right	right subscapular region, between latissismus and serratus muscle	9x7x2.5	pain	none	1.5 years	complete marginal resection	no	hematoma, seroma	none
7	79	F	housewife	right	left subscapular region, between latissismus and serratus muscle	6.5x2.5x5	swelling dyspnea hypertension	arteriosclerosis	1 year	complete marginal resection	no	seroma	none, complete remission of dyspnea and hypertension

### Pathology

**Figure 3:**
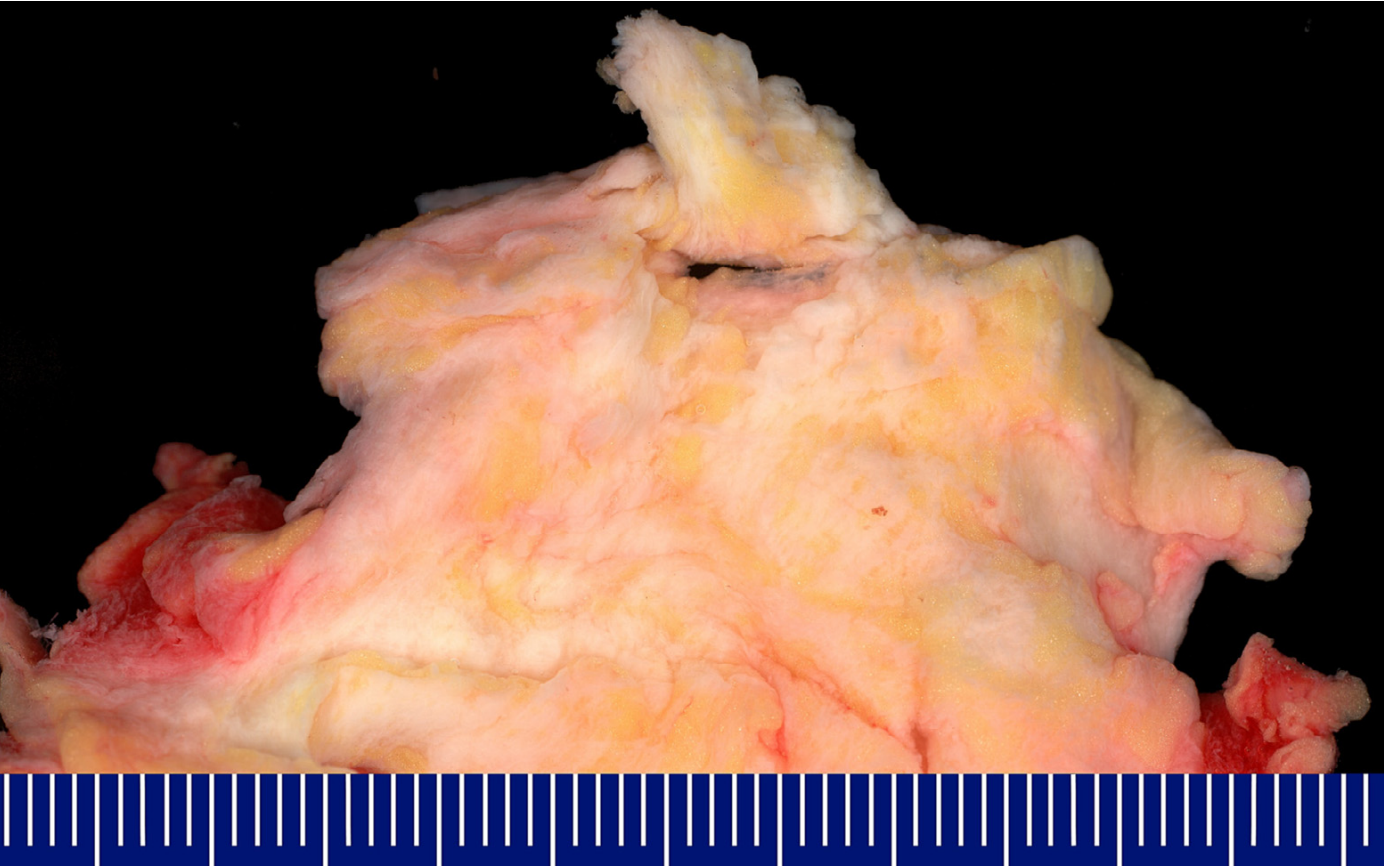
Macroscopic aspect of elastofibroma dorsi: Poorly defined fibroelastotic tumor with entrapment of fatty remnants.

**Figure 4:**
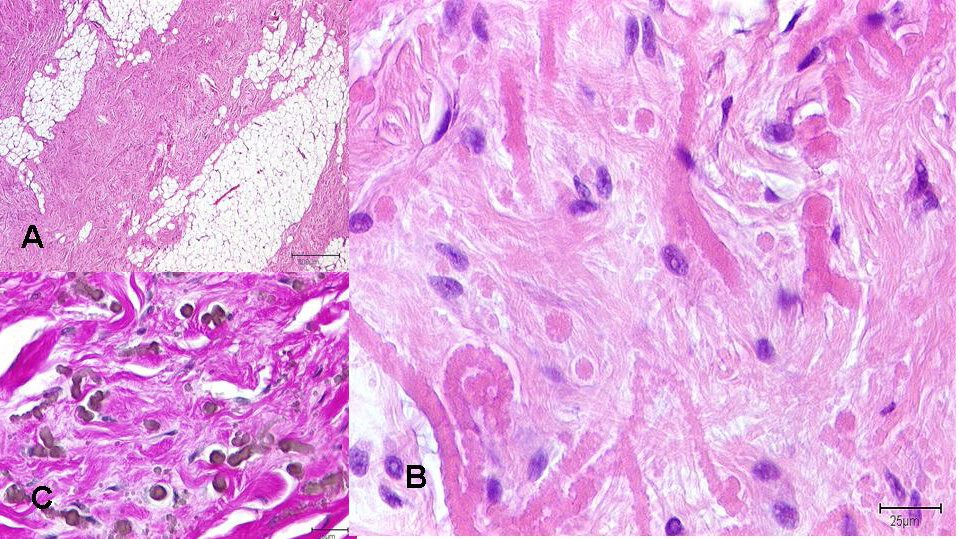
Microscopic findings in elastofibroma dorsi: 4a): Fibrous, collagenous strands intermingled with fat cells (hematoxylin-eosine-staining). 4b): Collagenous material and roundly shaped elastic fibres, mesenchymal cells with bland nuclei (hematoxylin-eosine-staining). 4c): Elastic fibres and structures forming discs and globules stained dark brown to black using an elastic stain (Elastica-van-Gieson).

Macroscopic findings: The soft tissue lesions were typically characterized by an irregular, poorly defined fibroelastotic mass with a slightly rubbery, elastic consistence. The cut surface showed strands of white and yellow tissue caused by the entrapment of fatty remnants, similar to a "checkerboard" pattern (figure 3). The tumours were not encapsulated. Microscopic findings: Histologically, all tumors were composed of fibrous, collagenous strands and plump, sometimes elongated mostly round-shaped elastic fibres which were densely packed. The elastic structures typically formed discs or globules and sometimes appeared in an "asbestos-body-like" fashion. These fibres were difficult to detect using hematoxylin-eosine-staining, especially during the frozen section procedure. They were best highlighted using elastic stain (Elastica-van-Gieson) which stained the fibres dark brown to black. The lesions were predominantly hypocellular with fibrocytic and fibroblastic cells without atypia and mitotic activity (figure 4a–c).

## Discussion

The pathogenesis of elastofibroma dorsi is still unclear, but repetitive microtrauma caused by friction between the scapula and the thoracic wall may cause reactive hyperproliferation of fibroelastic tissue [9-13]. A systematic review of the literature gave no further hints to the role of microtraumatization because most authors did not provide any information about their patients' activity. There is a striking predominance of the female gender from 5:4 to 13:1, depending on the study, suggesting that microtrauma alone cannot be the major factor in genesis of this lesion [1-4]. Previous publications referred to other sites of friction exposure as the tricuspid valve, axilla, foot, and ischial tuberosity; however, other reports of less common sites of manifestation with lower mechanical stress like the mediastinum, the stomach, the greater omentum, the inguinal region, the orbita, and the intraspinal space support this theory [3,11,14-18]. With the tumors occurring at the dominant and nondominant hand site in our patients, there seemed to be no association to mechanic stress assuming the dominant side was exposed to a higher level of repetitive microtrauma during lifetime. Additionally, only one of our patients had a history of extensive physical activity in his life (canoeist). Several authors proposed vascular insufficiency as a possible reason for the degenerative changes [8,12]. A familial predisposition with an underlying enzymatic defect may exist in 30%, but this has never been finally proved [2,19,20]. Large case series from Japan strongly suggest that hereditary factors may be a predisposition for this lesion [2,21]. The nature of the altered elastic fibres is disputed and controversial. They may be caused by abnormal elastogenesis or by degenerating as a secondary process, or even by a combination of both processes [8,13,20,22,23].

The symptoms of elastofibroma dorsi depend on the site and size of the lesion and may present as shoulder pain or snapping scapula as in our patients. In 50% of the cases, the tumor remains asymptomatic or causes mild discomfort only, explaining the long periods of up to 67 years between the onset of the symptoms and treatment [1-4,24,25]. Large lesions may simulate scapula alata, by elevating the scapula. If palpable, the tumor may mimic semimobility due to its elastic fibres, but intraoperatively it normally shows adherent to the surrounding tissue. It occurs predominantly at the right side but, in up to 50% of the cases, it is found bilaterally [8]. In our collective, this proportion was 14%. The coincidence of hypertension and dyspnea with elastofibroma has not yet been described and may be unrelated, whereas a large tumor may disturb thoracic elasticity and movements and therefore could cause dyspnea by interfering with the breathing motor function.

Aside from a possible soft tissue signal intensity or elevated scapula, plain radiographs do not show specific changes. On MRI, probably the most reliable non-invasive technique in diagnosis, the lesions mostly show a signal intensity, comparable to that of muscle, margins are well defined and signal intensity is mostly low. Interspersed adipose strands cause a heterogeneous structure with longitudinal areas of higher signal intensity [4,11,26-29]. In all of our patients the findings on MRI were consistent with the criteria mentioned above. After application of contrast agent, normally faint but also marked enhancement mimicking malignancy may be observed [30-32]. CT shows the same changes but is less sensitive for visualizing the strands of fatty tissue [29]. On PET-CT radiotracer accumulation of the hypermetabolic tumor has been described [33]. Differential diagnosis includes sarcomas, aggressive fibromatosis, lipoma, and fibroma. Ultrasound patterns of the tumor are characteristic including fasciculated structures with hypo- and hyperechogeneous striae of different thickness similar to that of muscle tissue but less organized. Colour Doppler shows vascularization patterns similar to the surrounding muscle. In the hands of an experienced examiner, ultrasound may represent a quick and cheap diagnostic tool [34-36]. Due to its muscle like appearance in all of the imaging procedures mentioned, the lesion may go undiagnosed or, in case of abnormal features, misdiagnosed. The advanced age of the patients, the typical localization, female gender or bilateral manifestation support the presumptive diagnosis of elastofibroma. In these cases and with clear imaging findings, one may refrain from biopsy. In all other cases, in contrast to other authors [26,28,37-40], we strongly recommend that tumor material be obtained to confirm the presumptive or to establish another diagnosis, because MRI, CT or ultrasound and clinical findings cannot give final safety [1,2,32,41-45]. Fine needle aspiration [46] is not recommended because of the inherent hypocellularity of the tumor. An open biopsy or at least a core needle biopsy should be performed to get a representative tissue specimen. Histomorphologically, the diagnosis is based on the presence of the altered elastic fibres embedded in a collagenous matrix, riddled with various amounts of fat cells. These elastic fibres are often fragmented into discs or globules and larger than regular ones [13,20,47]. Ultrastructurally, the elastinophilic material frequently contains a central core of mature elastic tissue and appears to be secreted by active fibroblasts; this further substantiates the thesis that the elastic material in elastofibroma is derived from excessive production by fibroblasts rather than from elastotic degeneration of collagen. Dense granular bodies within the fibroblast cytoplasm are described, which are thought to represent elastin or elastin precursors [48].

In incidental diagnosis of asymptomatic lesions there is no need for excision as malignant transformation has never been described. Only in cases of discomfort, snapping or blocking scapula and pain, marginal resection is widely recommended according to the psychological and physical strain of the patient [1,2,49], but anecdotal reports mentioned good results with radiotherapy as well [16,17]. This may be an option especially for manifestations in unresectable locations. The high incidence of seromas in our patients, whereas there is no report about seromas in the literature, may be a result of insufficient immobilisation. Taking into account the usually advanced age of the patients, immobilisation bears the risk of remaining stiffness in the shoulder girdle, whereas punctuation of a seroma may only prolong reconvalescence and cause mild discomfort. Our patients retrospectively did not experience postoperative seroma as relevant discomfort. All patients were free of the disease at follow-up time, concurring with the literature reporting only a few cases of recurrence [2,32,50].

## Conclusion

In differential diagnosis of soft tissue tumors located at the infrascapular region, elastofibroma should be considered as likely diagnosis by the surgeon and the surgical pathologist. We prefer MRI to localize and identify the lesion. In elderly patients or patients with bilateral manifestation and definite findings in imaging, it may be justified to refrain from biopsy. Otherwise, open biopsy should be performed to exclude malignancy and to reassure the asymptomatic patient that no surgical treatment is necessary. Unnecessary wide and radical resections in the symptomatic patient can be avoided because marginal resection has proven to be sufficient. We recommend postoperative wound drainage and compression garment, as well as shoulder immobilization for one week to reduce postoperative seroma.

## Competing interests

The author(s) declare that they have no competing interests.

## Authors' contributions

**AD **conceptualised the study, gathered the data and wrote the manuscript.

**PV **analysed and interpreted the data.

**KB **acquired and weighed the data.

**WP **evaluated the MRI findings and edited the radiology section.

**DW **was involved in drafting the manuscript and critically revising it.

**ML **interpreted the data and revised the manuscript.

**LS **reviewed the literature and analysed the data.

**HS **initiated the study and supervised the process. He gave final approval for publication.

**CK **performed the histopathological evaluation and interpretation of the data.

## References

[B1] Briccoli A, Casadei R, Di Renzo M, Favale L, Bacchini P, Bertoni F (2000). Elastofibroma dorsi. Surg Today.

[B2] Nagamine N, Nohara Y, Ito E (1982). Elastofibroma in Okinawa. A clinicopathologic study of 170 cases. Cancer.

[B3] Naylor MF, Nascimento AG, Sherrick AD, McLeod RA (1996). Elastofibroma dorsi: radiologic findings in 12 patients. AJR Am J Roentgenol.

[B4] Oueslati S, Douira-Khomsi W, Bouaziz MC, Zaouia K (2006). Elastofibroma dorsi: A report on 6 cases. Acta Orthop Belg.

[B5] Marin ML, Perzin KH, Markowitz AM (1989). Elastofibroma dorsi: benign chest wall tumor. J Thorac Cardiovasc Surg.

[B6] Brandser EA, Goree JC, El-Khoury GY (1998). Elastofibroma dorsi: prevalence in an elderly patient population as revealed by CT. AJR Am J Roentgenol.

[B7] Giebel GD, Bierhoff E, Vogel J (1996). Elastofibroma and pre-elastofibroma--a biopsy and autopsy study. Eur J Surg Oncol.

[B8] Jarvi OH, Lansimies PH (1975). Subclinical elastofibromas in the scapular region in an autopsy series. Acta Pathol Microbiol Scand [A].

[B9] Jarvi O, Saxen E (1961). Elastofibroma dorsi. Acta Pathol Microbiol Scand.

[B10] Machens HG, Mechtersheimer R, Gohring U, Schlag PN (1992). Bilateral elastofibroma dorsi. Ann Thorac Surg.

[B11] Hoffman JK, Klein MH, McInerney VK (1996). Bilateral elastofibroma: a case report and review of the literature. Clin Orthop Relat Res.

[B12] Stemmermann GN, Stout AP (1962). Elastofibroma dorsi. Am J Clin Pathol.

[B13] Winkelmann RK, Sams WM (1969). Elastofibroma. Report of a case with special histochemical and electron-microscopic studies. Cancer.

[B14] De Nictolis M, Goteri G, Campanati G, Prat J (1995). Elastofibrolipoma of the mediastinum. A previously undescribed benign tumor containing abnormal elastic fibers. Am J Surg Pathol.

[B15] Renshaw TS, Simon MA (1973). Elastofibroma. J Bone Joint Surg Am.

[B16] Prete PE, Henbest M, Michalski JP, Porter RW (1983). Intraspinal elastofibroma. A case report. Spine.

[B17] Deutsch GP (1974). Elastofibroma dorsalis treated by radiotherapy. Br J Radiol.

[B18] Mohan JC, Goel PK, Gambhir DS, Khanna SK, Arora R (1987). Calcified mobile papillary fibroelastoma of the tricuspid valve: a case report. Indian Heart J.

[B19] Enjoji M, Sumiyoshi K, Sueyoshi K (1985). Elastofibromatous lesion of the stomach in a patient with elastofibroma dorsi. Am J Surg Pathol.

[B20] Fukuda Y, Miyake H, Masuda Y, Masugi Y (1987). Histogenesis of unique elastinophilic fibers of elastofibroma: ultrastructural and immunohistochemical studies. Hum Pathol.

[B21] Sakae K (1973). Elastofibroma dorsi: Clinicopathological and electron microscope studies. Acta Med Univ Kagoshimaensis.

[B22] Akhtar M, Miller RM (1977). Ultrastructure of elastofibroma. Cancer.

[B23] Jarvi OH, Saxen AE, Hopsu-Havu VK, Wartiovaara JJ, Vaissalo VT (1969). Elastofibroma--a degenerative pseudotumor. Cancer.

[B24] Majo J, Gracia I, Doncel A, Valera M, Nunez A, Guix M (2001). Elastofibroma dorsi as a cause of shoulder pain or snapping scapula. Clin Orthop Relat Res.

[B25] Greenberg JA, Lockwood RC (1989). Elastofibroma dorsi. A case report and review of the literature. Orthop Rev.

[B26] Yu JS, Weis LD, Vaughan LM, Resnick D (1995). MRI of elastofibroma dorsi. J Comput Assist Tomogr.

[B27] Soler R, Requejo I, Pombo F, Saez A (1998). Elastofibroma dorsi: MR and CT findings. Eur J Radiol.

[B28] Massengill AD, Sundaram M, Kathol MH, el-Khoury GY, Buckwalter JH, Wade TP (1993). Elastofibroma dorsi: a radiological diagnosis. Skeletal Radiol.

[B29] Malghem J, Baudrez V, Lecouvet F, Lebon C, Maldague B, Vande Berg B (2004). Imaging study findings in elastofibroma dorsi. Joint Bone Spine.

[B30] Schick S, Zembsch A, Gahleitner A, Wanderbaldinger P, Amann G, Breitenseher M, Trattnig S (2000). Atypical appearance of elastofibroma dorsi on MRI: case reports and review of the literature. J Comput Assist Tomogr.

[B31] Zembsch A, Schick S, Trattnig S, Walter J, Amann G, Ritschl P (1999). Elastofibroma dorsi. Study of two cases and magnetic resonance imaging findings. Clin Orthop Relat Res.

[B32] Kransdorf MJ, Meis JM, Montgomery E (1992). Elastofibroma: MR and CT appearance with radiologic-pathologic correlation. AJR Am J Roentgenol.

[B33] Pierce JC, Henderson R (2004). Hypermetabolism of elastofibroma dorsi on PET-CT. AJR Am J Roentgenol.

[B34] Cota C, Solivetti F, Kovacs D, Cristiani R, Amantea A (2006). Elastofibroma dorsi: histologic and echographic considerations. Int J Dermatol.

[B35] Bianchi S, Martinoli C, Abdelwahab IF, Gandolfo N, Derchi LE, Damiani S (1997). Elastofibroma dorsi: sonographic findings. AJR Am J Roentgenol.

[B36] Solivetti FM, Bacaro D, Di Luca Sidozzi A, Cecconi P (2003). Elastofibroma dorsi: ultrasound pattern in three patients. J Exp Clin Cancer Res.

[B37] Baudrez V, Malghem J, Van de Berg B, Lebon C, Lecouvet F, Maldague B (1998). [Ultrasonography of dorsal elastofibroma. Apropos of 6 cases]. J Radiol.

[B38] Gould ES, Javors BR, Morrison J, Potter H (1989). MR appearance of bilateral periscapular elastofibromas. J Comput Assist Tomogr.

[B39] Kudo S (2001). Elastofibroma dorsi: CT and MR imaging findings. Semin Musculoskelet Radiol.

[B40] Nishida A, Uetani M, Okimoto T, Hayashi K, Hirano T (2003). Bilateral elastofibroma of the thighs with concomitant subscapular lesions. Skeletal Radiol.

[B41] Pisharodi LR, Cary D, Bernacki EG (1994). Elastofibroma dorsi: diagnostic problems and pitfalls. Diagn Cytopathol.

[B42] Berthoty DP, Shulman HS, Miller HA (1986). Elastofibroma: chest wall pseudotumor. Radiology.

[B43] Ghiatas AA, Armstrong S, Tio FO (1989). Case report 583: Elastofibroma dorsi. Skeletal Radiol.

[B44] Lang P, Suh KJ, Grampp S, Steinbach L, Steiner E, Peterfy C, Tirman P, Schwickert H, Rosenau W, Genant HK (1995). [CT and MRI in elastofibroma. A rare, benign soft tissue tumor]. Radiologe.

[B45] Liessi G, Tregnaghi A, Barbazza R, Scapinello A, Muzzio PC (1991). Elastofibroma: CT and MR findings. J Belge Radiol.

[B46] Domanski HA, Carlen B, Sloth M, Rydholm A (2003). Elastofibroma dorsi has distinct cytomorphologic features, making diagnostic surgical biopsy unnecessary: cytomorphologic study with clinical, radiologic, and electron microscopic correlations. Diagn Cytopathol.

[B47] Nakamura Y, Ohta Y, Itoh S, Haratake A, Nakano Y, Umeda A, Shima H, Tomoda N (1992). Elastofibroma dorsi. Cytologic, histologic, immunohistochemical and ultrastructural studies. Acta Cytol.

[B48] Dixon AY, Lee SH (1980). An ultrastructural study of elastofibromas. Hum Pathol.

[B49] Schafmayer C, Kahlke V, Leuschner I, Pai M, Tepel J (2006). Elastofibroma dorsi as differential diagnosis in tumors of the thoracic wall. Ann Thorac Surg.

[B50] McGregor JC, Rao SS (1974). Elastofibroma: a rare cause of painful shoulder. Br J Surg.

